# Disruption of circadian timing increases synaptic inhibition and reduces cholinergic responsiveness in the dentate gyrus

**DOI:** 10.1002/hipo.23301

**Published:** 2021-01-13

**Authors:** Laura McMartin, Marianna Kiraly, H. Craig Heller, Daniel V. Madison, Norman F. Ruby

**Affiliations:** 1 Department of Molecular and Cellular Physiology, Stanford University, Stanford, California; 2 Biology Department, Stanford University, Stanford, California

**Keywords:** acetylcholine, carbachol, dentate, hippocampus, sex differences, Siberian hamster

## Abstract

We investigated synaptic mechanisms in the hippocampus that could explain how loss of circadian timing leads to impairments in spatial and recognition memory. Experiments were performed in hippocampal slices from Siberian hamsters (*Phodopus sungorus*) because, unlike mice and rats, their circadian rhythms are easily eliminated without modifications to their genome and without surgical manipulations, thereby leaving neuronal circuits intact. Recordings of excitatory postsynaptic field potentials and population spikes in area CA1 and dentate gyrus granule cells revealed no effect of circadian arrhythmia on basic functions of synaptic circuitry, including long-term potentiation. However, dentate granule cells from circadian-arrhythmic animals maintained a more depolarized resting membrane potential than cells from circadian-intact animals; a significantly greater proportion of these cells depolarized in response to the cholinergic agonist carbachol (10 μM), and did so by increasing their membrane potential three-fold greater than cells from the control (entrained) group. Dentate granule cells from arrhythmic animals also exhibited higher levels of tonic inhibition, as measured by the frequency of spontaneous inhibitory postsynaptic potentials. Carbachol also decreased stimulus-evoked synaptic excitation in dentate granule cells from both intact and arrhythmic animals as expected, but reduced stimulus-evoked synaptic inhibition only in cells from control hamsters. These findings show that loss of circadian timing is accompanied by greater tonic inhibition, and increased synaptic inhibition in response to muscarinic receptor activation in dentate granule cells. Increased inhibition would likely attenuate excitation in dentate-CA3 microcircuits, which in turn might explain the spatial memory deficits previously observed in circadian-arrhythmic hamsters.

## 1 | INTRODUCTION

There is an extensive research literature showing that disruptions in the circadian timing system leads to substantial deficits in learning and memory processes (for detailed reviews see Smarr, Jennings, Driscoll, & Kriegsfeld, 2014, and Krishnan & Lyons, 2015). Among rodents, one of the most consistent findings from this body of research is that circadian dysfunction impairs performance on memory tests that involve hippocampal encoding. This is evidenced mainly by experiments in which circadian timing is disrupted by simulated time zone transitions (jet-lag) that impair performance on tests such as passive avoidance, water maze, fear conditioning, and alternation behavior.

In humans, circadian rhythm disruption can impair cognitive performance (Molzof, Prapanjaroensin, Patel, Gamble, & Patrician, 2019), and clinical studies have suggested that age-related declines in circadian timing may contribute to memory deficits among the elderly (Schlosser Covell et al., 2012; Stranahan, 2012; Tranah et al., 2011; van Someren et al., 1996), an effect that appears to be mediated by the hippocampus (Sherman, Mumford, & Schnyer, 2015). Several authors have also promoted the idea that cognitive decline associated with aging may be slowed or even improved by rescuing circadian function (Coogan et al., 2013; Stranahan, 2012; van Someren et al., 1996; Wu & Swaab, 2007). Our own studies in animals have shown that a complete loss of circadian timing severely impaired spatial and recognition memory (Fernandez et al., 2014; Ruby et al., 2008; Ruby et al., 2013). Memory was, however, restored by a daily regimen of scheduled meal timing (Ruby et al., 2017). Thus, memory impairments were rescued by imposing a daily rhythm on the animals’ behavior. The goal of this study was to identify synaptic mechanisms in the hippocampus that could explain how circadian dysfunction leads to memory deficits.

We performed these experiments in hippocampal slices prepared from Siberian hamsters (*Phodopus sungorus*) because unlike mice and rats, these animals can be rendered circadian-arrhythmic without the need for genetic or surgical manipulations to silence the circadian system, thus leaving neuronal circuits intact (Grone et al., 2011; Ruby et al., 2008). These animals are unique in that circadian timing can be completely eliminated by a one-time brief photic treatment (i.e., the disruptive phase shift [DPS] protocol described in methods). This simple treatment completely eliminates circadian timing in the molecular clock in the core of the circadian system, the hypothalamic suprachiasmatic nucleus (SCN; Grone et al., 2011).

Siberian hamsters that have been rendered circadian-arrhythmic by the DPS protocol exhibit severe impairments in object recognition and spatial working memory (Ruby et al., 2008; Ruby et al., 2013). In a novel object recognition (NOR) test, arrhythmic hamsters could not recognize an object that they had explored 20 min earlier (Ruby et al., 2008). Spatial working memory, as evaluated by spontaneous alternation (SA) in a T-maze, revealed that control hamsters alternated at rates comparable to mice and rats (>75%; Deacon & Rawlins, 2006), whereas circadian-arrhythmic animals made random arm choices (Ruby et al., 2013). These results suggested that circadian arrhythmia originating in the SCN interfered with information processing in the hippocampus. This hypothesis was confirmed by eliminating circadian timing with the DPS protocol and then surgically ablating the SCN of those animals. The surgery removed the deficit and restored performance on the NOR and SA tests of DPS-treated hamsters to the same levels as the control animals (Fernandez et al., 2014).

In the present study, we evaluated the impact of circadian arrhythmia on several parameters of hippocampal synaptic physiology in in vitro slices of hippocampal area CA1 and the dentate gyrus. We then focused on cholinergic signaling in the dentate gyrus because of the well-established role of cholinergic projections from the medial septum/diagonal band to the granule cells in the dentate, which are critically necessary for alternation behavior and for spatial working memory (Dember & Richman, 1989; Haam & Yakel, 2017; Kesner, 2007; Khakpai, Nasehi, Haeri-Rohani, Eidi, & Zarrindast, 2013; Sasaki et al., 2018). We found that the basic function of CA1 and dentate synaptic circuits were unaltered, but that dentate cells from circadian-arrhythmic animals exhibited higher levels of spontaneous inhibition. The cholinergic agonist carbachol produced a higher net level of spontaneous and evoked inhibition in dentate granule cells from circadian-arrhythmic hamsters compared to entrained ones. We propose that this increased inhibition impaired spatial memory by reducing excitation-and therefore spatial memory encoding-in the dentate-CA3 circuit. This is the first study to identify a synaptic mechanism that can potentially explain how circadian dysfunction impairs memory processing in the hippocampus.

## 2 | MATERIALS AND METHODS

### 2.1 | Animals and housing conditions

Male and female Siberian hamsters (*Phodopus sungorus*) were bred in the laboratory as described previously (Ruby et al., 2013). All experimental procedures were approved by Stanford University’s Administrative Panel on Laboratory Animal Care (Animal Use Protocol #14988) and were conducted in accordance with the NIH Guide for the Care and Use of Laboratory Animals. During each experiment, animals were housed individually; locomotor activity was measured by passive infrared motion detectors. Activity bouts were summed in 10-min intervals and stored on computer.

### 2.2 | Induction of circadian arrhythmia (the disruptive phase shift [DPS] protocol)

Males and females were 2–3 months of age when the DPS treatment was administered. Fourteen days after being housed singly (16 hr light: 8 hr dark/day), lights in the activity recording chambers were turned on for 2 hr beginning 5 hr after lights-off (i.e., a 2-hr light pulse). On the next day, the LD cycle was phase delayed by 3 hr. Animals remained in the 16:8 LD cycle thereafter. Hamsters with confirmed loss of circadian locomotor rhythms (i.e., arrhythmic, ARR) at 4–8 weeks after the DPS treatment were randomly assigned to their experimental groups, along with age- and gender-matched controls from the hamster colony (i.e., entrained, ENT). Loss of circadian timing in ARR hamsters was confirmed by periodogram analysis (*p* < .01).

### 2.3 | Preparation of acute hippocampal slices

Hippocampi were dissected from the brain and cut into 500 μm transverse sections using a manual razor blade tissue sectioner (Stoelting, Inc). Slices were cut in ice-cold oxygenated artificial cerebrospinal fluid (ACSF) containing (mM): 119 NaCI, 2.5 KCI, 6.0 MgSO_4_, 2.5 CaCI_2_, 1.0 NaH_2_PO_4_, 26.2 NaHCO_3_, and 11.0 glucose followed by a 2-hr recovery period in ACSF at room temperature in an interface storage chamber, with the upper surface of the slices exposed to humidified 95%O_2_/5%CO_2_ (carbogen) gas. Slices were moved individually to a recording chamber where they were submerged beneath a continuous flow of 95% O_2_/5% CO_2_ ACSF at 30°C.

### 2.4 | Electrophysiology

Synaptic field potentials (fEPSPs) were recorded with 3 M NaCL-filled glass micropipettes (tip resistance ~2 MΩ) in stratum radiatum and population spikes were recorded in s. pyramidale, both in area CA1, in response to Schaffer collateral stimulation. In dentate gyrus, stimulation was applied in proximal perforant path, while fEPSPs were recorded in more distal mid-s. moleculare. In dentate, we did not attempt to differentiate between medial and lateral perforant paths; both electrodes were placed approximately half the distance between the granule cell layer and the hippocampal fissure, in their respective positions along the path. Recordings of populations spikes were performed simultaneously with the dendritic field, with the 2nd recording electrode placed in the granule cell layer, immediately below the recording electrode in s. moleculare. Stimuli of 100 μs duration were delivered through insulated stainless steel concentric microelectrodes (Frederick Haer, Inc).

When measuring the population spike in this study, we chose to measure the area of the spike, rather than the amplitude. This measurement was made by drawing an imaginary line from the field source peaks immediately before and after the spike, and then measuring the amplitude of each sampled data point (one every 100 μs at a sample rate of 10 kHz), and then summating those individual point amplitude measurements, essentially integrating the area of the spike. We chose this method because it is the simplest measure of the total postsynaptic neuron excitation that occurs with a stimulus. A population spike is essentially the summated extracellular currents from every individual stimulus-evoked neuron action potential generated within the range of the recording electrode. The more commonly-used measure of population spike amplitude is subject to both the number of cells excited and the synchrony of that excitation. For example, a narrow, high amplitude population spike, and a broad, low amplitude population spike can have the same area; the same number of excited postsynaptic neurons contributing, but at different synchrony. Thus, the more traditional amplitude measurements can be misleading for the purpose of these experiments, which was to have a measure of total excitation in the stimulated population of postsynaptic neurons.

We also assayed long-term potentiation (LTP) in both CA1 and dentate gyrus, using a tetanic stimulation protocol (100 Hz for 1 s, 4 times, 15 s apart). LTP experiments in the dentate gyrus were performed with a low concentration (10 μM) of picrotoxin in the ACSF to facilitate LTP induction. Experiments assaying inhibitory postsynaptic potentials (IPSPs), both evoked and spontaneous, were performed using whole-cell recording in the extracellular presence of 10 μM 6,7-dinitroquinoxaline-2,3-dione (DNQX) to block AMPA/Kainate-mediated excitatory transmission. The electrode internal solution, containing high-chloride to reverse and increase IPSP driving force for better detection, consisted of (mM): 120 KCL, 40 HEPES, 2 Mg-ATP, 0.317 Na-GTP, and 10.7 MgCI_2_. We recorded both stimulus-evoked IPSCs, generated by stimuli in the perforant path, and spontaneous IPSC, which result from spontaneous firing of inhibitory interneurons. Carbachol was applied in the superfusing ACSF at a concentration of 10 μM. For stimulus-evoked IPSPs, and because these experiments were performed in the presence of the AMPA receptor blocker DNQX (10 μM), we placed the stimulating electrode close enough to the recording electrode to evoke monosynaptic evoked IPSPs (Davies, Davies, & Collingridge, 1990) and set the stimulus strength just above the current that produced a maximal monosynaptic IPSP. The amplitude of stimulus artifacts in all raw electrophysiology traces shown in figures were truncated for illustration purposes.

### 2.5 | Data analysis

For most analyses, data from individual cells were averaged within each animal from which they were obtained so that each data point represents a single animal. Analyses where data were grouped by cells, and not by animals, are explicitly stated in the text. Statistical comparisons were made using either ANOVA or repeated measures ANOVA to test for main effects (i.e., drug, rhythm status, sex) as indicated in the text. Subsequent pairwise comparisons were made using Sidak’s post-hoc correction for multiple comparisons. Comparisons between groups of spontaneous IPSPs were performed on cumulative distributions of the data with Kolmogorov-Smirnov (KS) tests. Differences in proportions of categorical data were tested by Fishers exact test. The absence of circadian rhythms was confirmed by periodogram analysis where the level of significance was set to *p* < .001. The data that support the findings of this study are available from the corresponding author upon reasonable request.

## 3 | RESULTS

### 3.1 | Circadian arrhythmia

**FIGURE 1:**
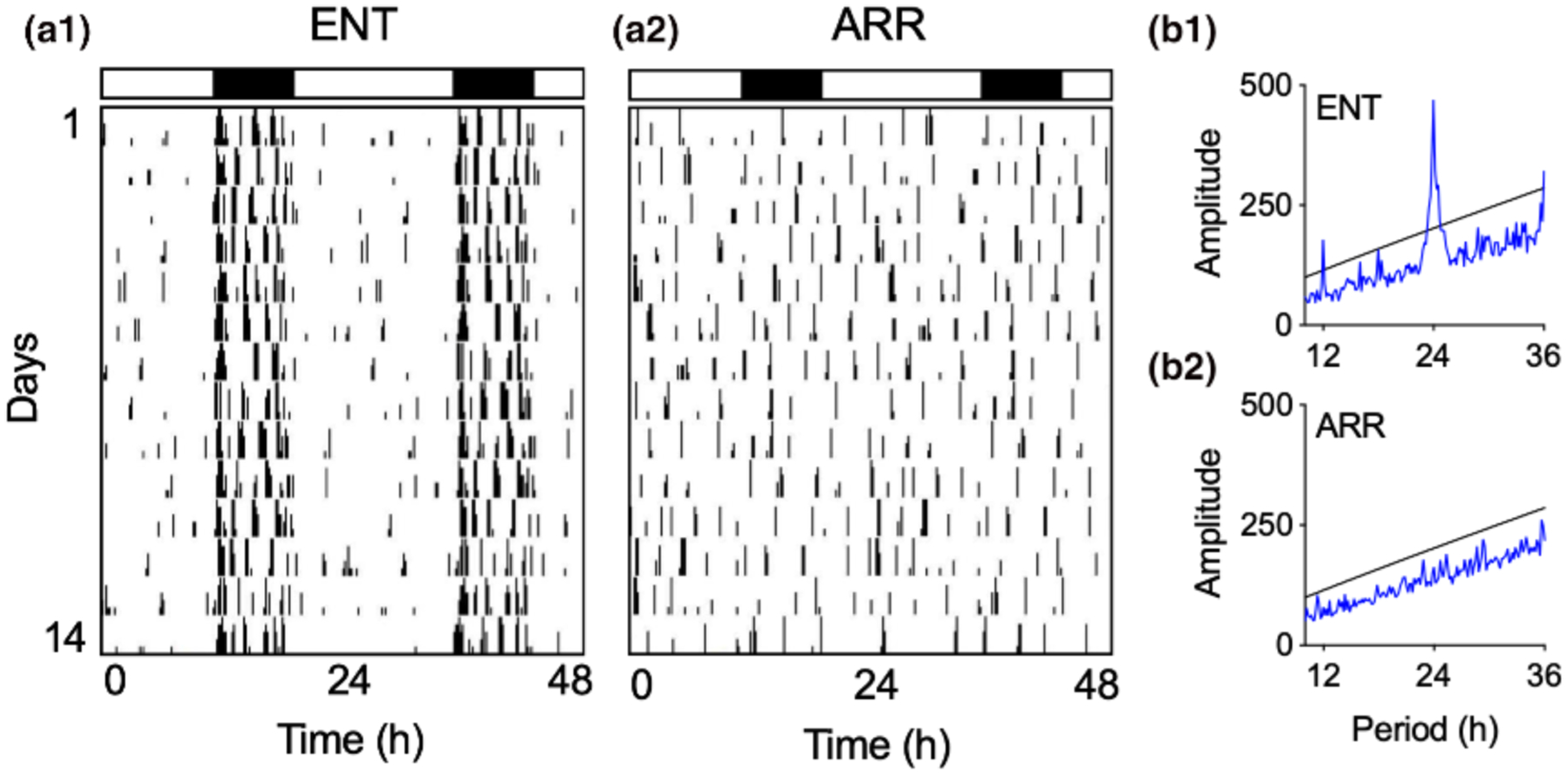
Representative examples of locomotor activity patterns that were used to designate hamsters as entrained (ENT, a1) or circadian-arrhythmic (ARR, a2). Vertical black hash marks indicate daily locomotor movement in the home cage and are double-plotted on a 48-hr time scale with data from consecutive days plotted from top to bottom. Most cage activity occurs at night for ENT hamsters as indicated by the black and white rectangles that indicate night (8 hr) and day (16 hr), respectively. By contrast, locomotor activity in ARR hamsters is distributed equally across 24 hr, indicating the loss of circadian timing. These observations were confirmed by chi-square time series analysis showing a robust daily rhythm with a peak at 24 hr with harmonics at 12 and 36 hr in ENT animals (b1), and a lack of periodicity in the ARR animal (b2)

Hippocampal slices were prepared between 4–8 weeks after the DPS treatment from hamsters that were determined to be circadian-arrhythmic and that were 3–5 months of age at the time tissue slices were made (ARR; Figure 1). Age-matched animals that had intact circadian rhythms and were entrained (ENT) to the environmental light-dark cycle served as control animals. A total of 115 hamsters (males and females) were used in this study. Our final sample sizes were: ENT males (38), ENT females (21), ARR males (34), ARR females (22).

### 3.2 | Input/output curves and paired-pulse facilitation

I/O curves and paired-pulse ratios are measurements commonly used to assay the basal state of hippocampal circuitry. I/O curves, which we constructed from the synaptic field potential response generated over a range of stimulus strengths, is generally taken to be an assay for whether a particular synaptic tract, such as the Schaffer collateral/CA1 or perforant path/dentate tracts, have a roughly normative number of synapses transmitting at a roughly normative strength. Pair-pulse ratios are generally taken as an index of presynaptic transmitter release, although as will be seen later in these results, they can also be influenced by postsynaptic factors. Long-term potentiation (LTP) is the widely-studied form of activity-dependent synaptic plasticity often thought to be involved in some way in the formation of memory.

**FIGURE 2:**
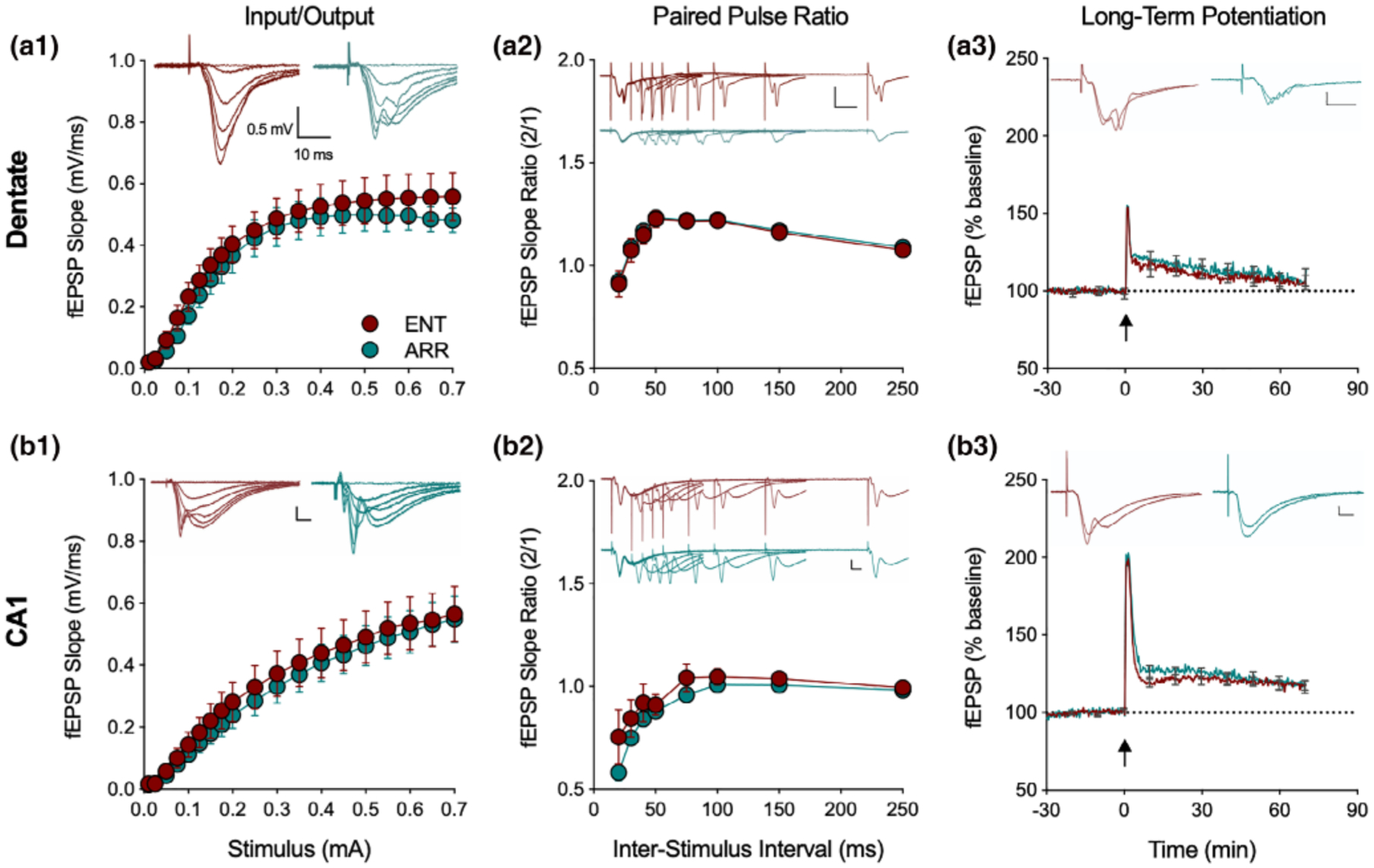
Loss of circadian timing did not affect basic excitatory circuit properties in the hippocampus. Recordings from the perforant path of the dentate gyrus of input/output curves (I/O, a1), paired pulse ratio (PPR, a2) and long-term potentiation (LTP, a3), with the same measures in the Schaffer collateral CA1 path of hippocampus (b1–3; *n* = 24 ENT, *n* = 23 ARR animals). There were no significant differences in these three measures between ENT (red) and ARR (green) either in dentate or CA1 (2-way ANOVA with repeated measures for stimulus, ISI, and time, *p* > .05). Inserts show representative traces at different stimulus intensities. Note that PPR and LTP responses are evaluated by their slopes, not amplitudes. Dotted lines in LTP panels indicate baseline values extrapolated past tetanus (arrow). All scale bars are 0.5 mV, 10 ms. Data expressed as means ± *SE*; for clarity, PPR only shown up to 250 ms, LTP error bars shown every 600 s

In general, we found no evidence for differences in the basal properties of synaptic transmission between hippocampal slices taken from ENT and ARR hamsters in any of these measures either in dentate gyrus or CA1 neurons (Figure 2). I/O curves of dendritic fEPSP responses, recorded over a range of stimulus strengths (0.01–0.7 mA) did not differ between ENT (*n* = 24) and ARR (*n* = 23) groups in the dentate gyrus (*p* > .05; stimulating electrode and recording electrode both placed in dentate molecular layer to assay perforant path transmission; Figure 2a1) nor in CA1 (*p* > .05; stimulating and recording electrodes both placed in stratum radiatum, stimulus near CA2 border and recording electrode mid-CA1; Figure 2b1). Likewise, we detected no change in the paired-pulse responses in the same recordings in dentate (*p* > .05; Figure 2a2) or CA1 (*p* > .05; Figure 2b2) over a range of inter-pulse intervals from 20 to 1,000 ms (20–250 ms shown in figures).

Comparison of LTP found no differences between ENT and ARR groups in baseline recordings (*p* > .05), in the perforant path/dentate (*p* > .05; Figure 2a3), or Schaffer collateral/CA1 (*p* > .05; Figure 2b3) synaptic pathways. We conclude from these results, that the loss of rhythms did not cause any detectable disruption in the basal properties of excitatory synaptic circuit function in dentate or CA1, nor did it reduce expression of the activity-dependent synaptic plasticity, LTP. Thus, no wholesale reorganization of dentate/hippocampal neural circuitry resulted from the abolishment of circadian rhythms in these animals; synaptic function within these networks was normal. Learning deficits in these animals are likely not attributable to an inhibition of LTP.

### 3.3 | Input/output curves and paired-pulse facilitation during carbachol treatment

We hypothesized that the memory deficits exhibited by ARR hamsters may be due to changes in cholinergic responsiveness in the neural circuitry of the hippocampus. This hypothesis is based on the observation that the SCN does not directly innervate the hippocampal formation, but innervates the ventral lateral septal nuclei, which in turn, influences cholinergic signaling from the medial septum to the dentate gyrus (i.e., the septohippocampal pathway; Krnjević & Ropert, 1981). Acetylcholine (Ach), and in particular the actions of Ach expressed through muscarinic receptors, have a number of actions on neurons and synapses of the hippocampus (Adams et al., 1982a, 1982b; Cole & Nicoll, 1984a, 1984b; Dannenberg, Young, & Hasselmo, 2017; Haam & Yakel, 2017; Halliwell & Adams, 1982; Madison, Lancaster, & Nicoll, 1987; Pitler & Alger, 1990, 1992; Vogt & Reghr, 2001). Thus, we tested the effects of the cholinergic agonist carbachol on I/O curves and PPF. We then focused on the dentate gyrus, an area with particularly prominent septal cholinergic input (Dutar, Bassant, Senut, & Lamour, 1995; Khakpai et al., 2013; Teles-Grilo Ruivo & Mellor, 2013), We conducted our analysis in field potentials recorded in the mid-molecular layer of the dentate to assay synaptic transmission as well as simultaneously in the granule cell layer to assay granule cell excitation, in response to perforant path stimulation.

**FIGURE 3:**
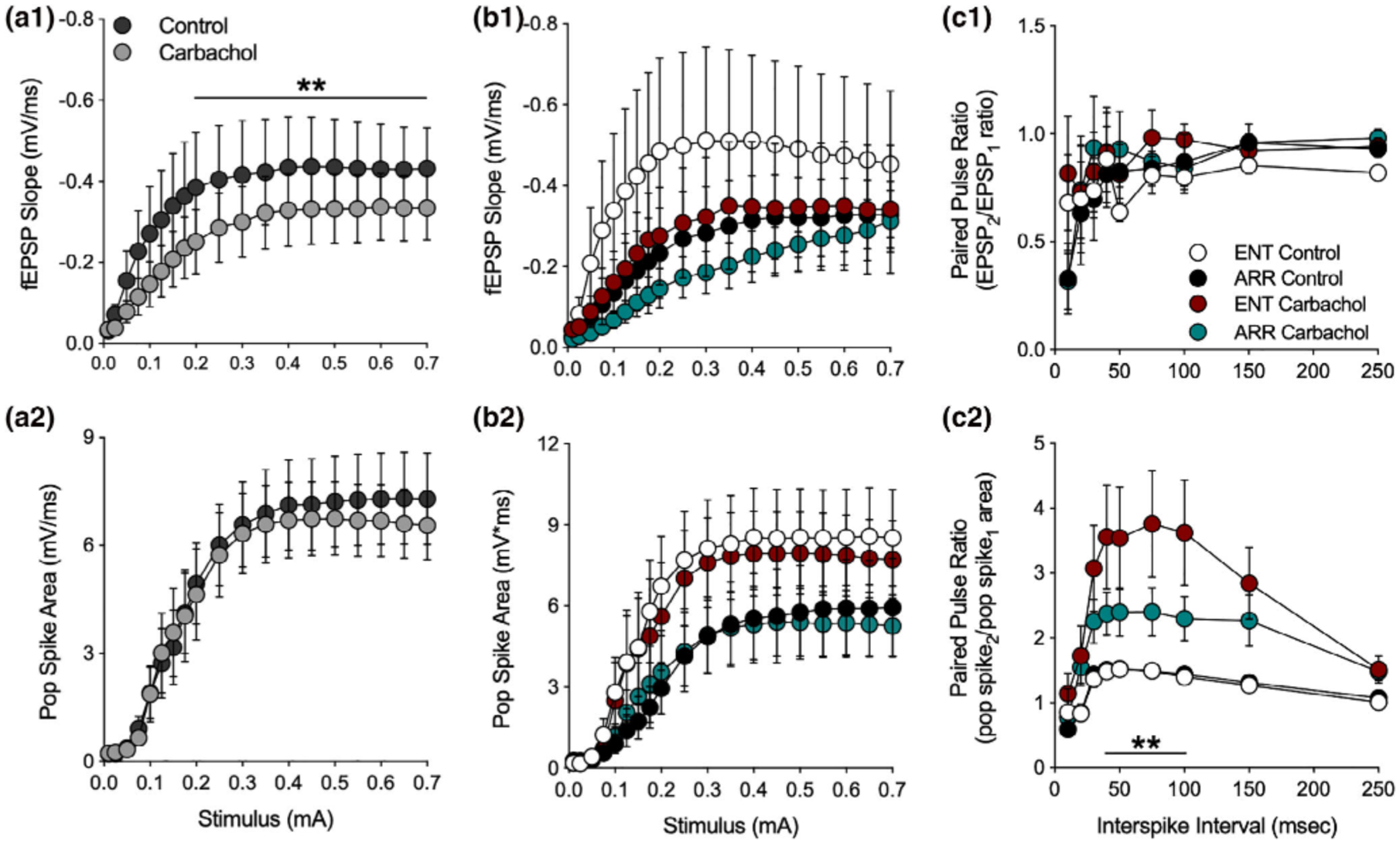
Effects of muscarinic receptor activation by carbachol in the dentate gyrus. Data are shown prior to (control) and during (carbachol) bath application of 10 mM carbachol. Stimulus-evoked fEPSPs (ENT and ARR combined, *n* = 22 animals) were suppressed by carbachol (a1) while simultaneously-recorded population spikes were unaffected (a2), however, there were no significant differences between ENT (*n* = 10) and ARR (*n* = 12) groups in these measures (b1, b2; *p* > .05). The high variance in the ENT control group (b1) was largely due to one cell, however, it was not a significant outlier (Grubbs’ test, *p* > .05). Paired pules ratio (PPR) of EPSPs was not affected by carbachol and did not differ between cells from ENT (*n* = 9) and ARR (*n* = 11) groups (c1; *p* > .05). By contrast, PPR of the population spike was significantly increased by carbachol in both ENT and ARR groups, although the effect was significantly reduced in cells from ARR animals (c2). ** Indicates stimulus (0.2–0.7 mA in panel c1), ISIs (40–100 ms in panel c2) that differed significantly between ENT and ARR groups under carbachol treatment (*p* < .01)

Bath application of carbachol (10 μM) caused a significant suppression of the I/O curve of fEPSPs in dentate (ENT and ARR combined) which is consistent with the action of muscarinic activation to suppress EPSPs in hippocampus or cortex (*F*_(1,21)_ = 21.2, *p* < .001; Figure 3a1; Dutar et al., 1995; Sheridan & Sutor, 1990; Kremin et al., 2006), but had no effect on the population spike (Figure 3a2). When these data were split into ENT and ARR groups, ANOVA confirmed that there was no significant effect of rhythm status (*p* > .05) or of carbachol (*p* > .05) on the fEPSP slope (Figure 3b1). The high variance in the ENT control group was largely due to one cell, however, it was not a significant outlier (Grubbs’ test, *p* > .05). Similar negative results were obtained for population spikes recorded simultaneously in the adjacent granule cell layer (Figure 3b2); there were no significant differences between ENT and ARR groups during baseline or carbachol conditions (*p* > .05), nor did carbachol suppress the population spike in ENT and ARR groups (*p* > .05; Figure 3b2).

These results are interesting because the magnitude of population spikes generally co-vary with the size of the EPSP that drives them. The fact that cholinergic suppression of the fEPSP was not accompanied by similar suppression of the population spike suggests with that the population spike is saturated, or that the coupling between the EPSP and the population spike (E-S coupling) is altered by carbachol. The second of these two possibilities is favored by comparison of the data in Figure 3b1,b2. Despite its action to suppress EPSPs, the paired pulse ratio of the fEPSP was not changed by the application of carbachol (*p* > .05; Figure 3c1), suggesting that presynaptic release probability remained unchanged by muscarinic receptor activation. By contrast, the PPF of the simultaneously recorded population spike was enhanced in the presence of carbachol (ENT and ARR combined, *F*_(1,38)_ = 13.9, *p* < .001; not shown), further supporting a mechanism downstream of the presynaptic excitatory transmitter release. But perhaps more interesting is the finding that this PPF of the population spike is significantly larger in slices from ENT animals than it is from ARR animals (Figure 3c2). Carbachol enhanced facilitation of the population spike PPF by approximately 140% in slices from ENT animals (*F*_(1,16)_ = 7.70, *p* = .013), but only by about 60% in ARR animals (*F*_(1,20)_ = 7.32, *p* = .014; Figure 3c2; intervals from 40–100 ms were significantly different between ENT and ARR, *p* < .01, Sidak’s). These results suggest that carbachol is enhancing facilitation of excitation via some indirect mechanism, perhaps a differential effect on synaptic inhibition.

### 3.4 | Inhibitory post synaptic potentials during carbachol treatment

**FIGURE 4:**
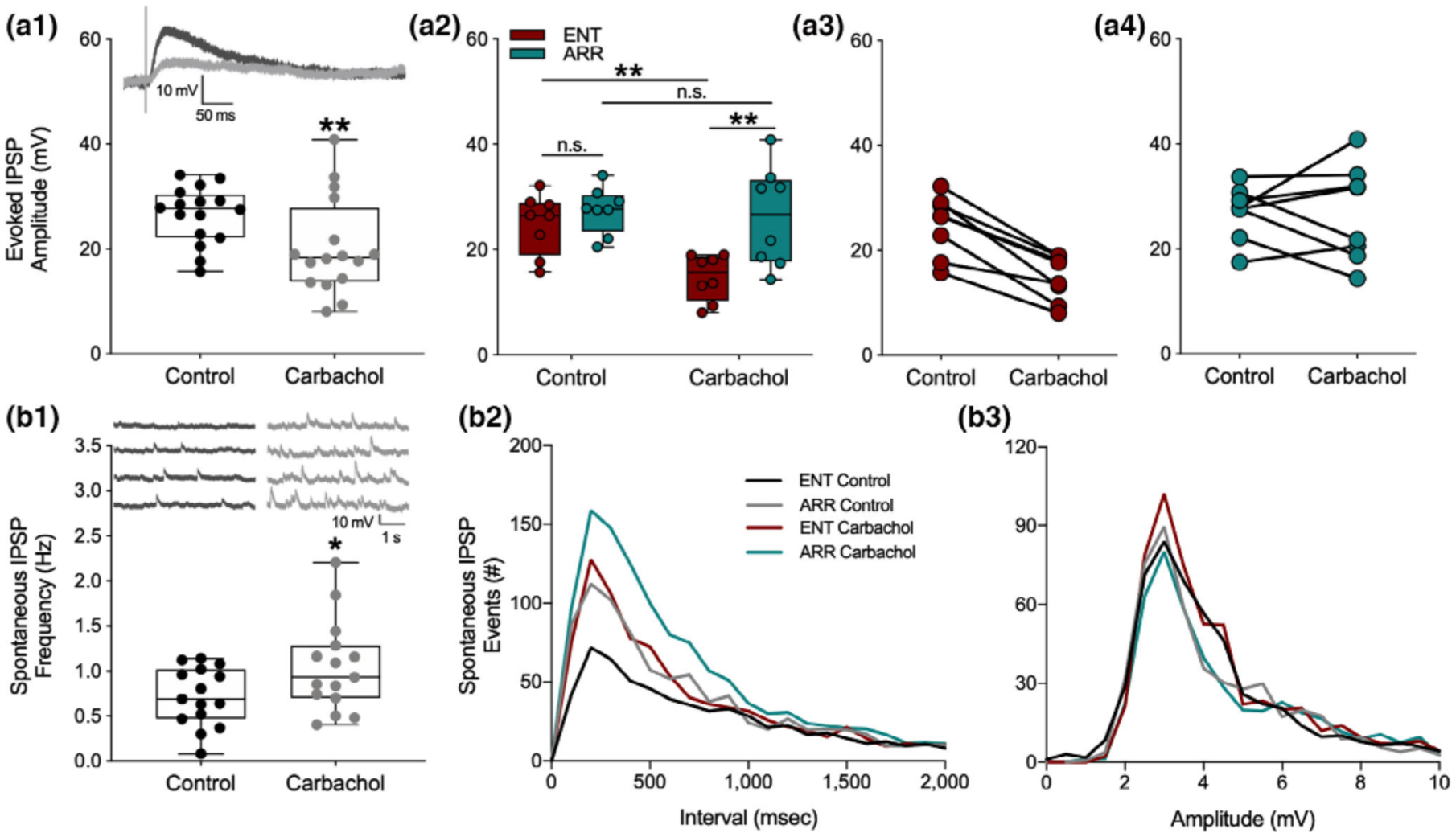
Synaptic inhibition induced by muscarinic receptor activation by carbachol was attenuated in dentate cells from circadian-arrhythmic animals. Carbachol application decreased stimulus evoked inhibitory transmission (a1; *n* = 18 cells, *p* < .001), but increased spontaneous tonic inhibition (b1; *p* = .020). IPSPs are depolarizing because the intracellular electrode solution contained a high concentration of chloride (see methods). Representative traces of inhibitory responses are shown in panels (a1) and (b1). Box plots show median values and extend from the 25th to 75th percentiles; whiskers extend to minimum and maximum data points with individual values shown as circles. Stimulus-evoked IPSPs were significantly suppressed only in cells from ENT animals (a2; *n* = 8 ENT, *n* = 7 ARR); amplitudes are shown for individual cells from ENT (a3) and ARR (a4) animals. Frequency distributions of spontaneous IPSP intervals (b2) and amplitudes (b3) during the first 150 s of recording. Statistical comparisons were made with KS tests from cumulative frequency distributions of the data in panels (b2) and (b3). Carbachol increased the number of spontaneous IPSP events in both ENT (*p* = .001) and ARR (*p* = .003) groups. Cells from ARR animals had significantly more IPSP events before (control; *p* = .014) and during (carbachol; *p* = .003) drug application compared to ENT (b2). There were no significant differences (*p* > .05) in amplitudes (b3) of spontaneous ISPS events. *p < .05, **p < .001, n.s. (nonsignificant difference)

An obvious potential explanation for the lack of suppression of population spikes in the face of EPSP suppression, as well as the increase in population spike PPF in the absence of fEPSP PPF, is that carbachol suppresses synaptic inhibition, which is targeting the soma of the neurons. This would both tend to move the population spike toward saturation, and also alter the E-S coupling between dendritic synaptic input and somatic excitation. Such an action would be consistent with some of the known actions of muscarinic receptor activation to suppress hippocampal inhibition (Bell, Bell, & McQuiston, 2013; McQuiston, 2014; McQuiston & Madison, 1999a, 1999b). To test for this, we recorded stimulus-evoked intracellularly-recorded monosynaptic inhibitory postsynaptic potentials (IPSPs) with whole cell electrodes in dentate granule cells. In these experiments, the stimulus strength was set just above the level needed to produce a maximal amplitude IPSP. As expected, carbachol caused a decrease in the size of evoked IPSPs (paired *t*-test, *p* = .003; Figure 4a1). However, at the same time, we detected an increase in the frequency of spontaneous IPSPs impinging on the granule cell (paired *t*-test, *p* = .020; Figure 4b1). These spontaneous IPSPs arise mostly as a result of spontaneous action potential discharge in inhibitory interneurons. In addition to being a source of tonic inhibition, they also serve as an index of the spontaneous firing rate of the inhibitory interneurons from which they arise (Bergles, Doze, Madison, & Smith, 1996; Doze, Cohen, & Madison, 1991).

To see if the effects of synaptic inhibition might be consistent with the differential effects of population spike PPF, we compared the intracellular measurements on synaptic inhibition between ENT and ARR groups. Carbachol significantly reduced stimulus-evoked IPSP amplitude (*F*_(1,13)_ = 14.69, *p* = .002), but only in ENT cells, and not in ARR (ENT: *p* = .005, ARR: *p* > .05; Sidak’s; Figure 4a2). There were no significant differences in stimulus-evoked IPSPs between ENT and ARR animals under control conditions (Sidak’s, *p* > .05, Figure 4a2).

Cumulative distributions were used to examine the effects of carbachol on the frequency and amplitude of IPSP events. In control recordings, the frequency of spontaneous IPSP events was significantly greater in the ARR group compared to ENT (KS test, *p* = .014; Figure 4b2). Application of carbachol increased the frequency of these events in both ENT and ARR slices (KS tests; ENT: *p* < .0001, ARR: *p* = .003; Figure 4b2), but the absolute frequency remained higher under carbachol in ARR cells compared to ENTs (KS test, *p* = .003; Figure 4b2). Differences between groups in spontaneous frequency of IPSPs were not accompanied by changes in the amplitude of these events (*p* > .05 for all KS tests of amplitude differences; Figure 4b3). Taken together, the consistently higher level of spontaneous IPSPs in ARR slices, along with the greater suppression of stimulus-evoked synaptic inhibition by carbachol in ENT, might well contribute to the lower levels of facilitation of granule cell population spike excitability observed in ARR compared to ENT groups.

### 3.5 | Membrane potential during carbachol treatment

**FIGURE 5:**
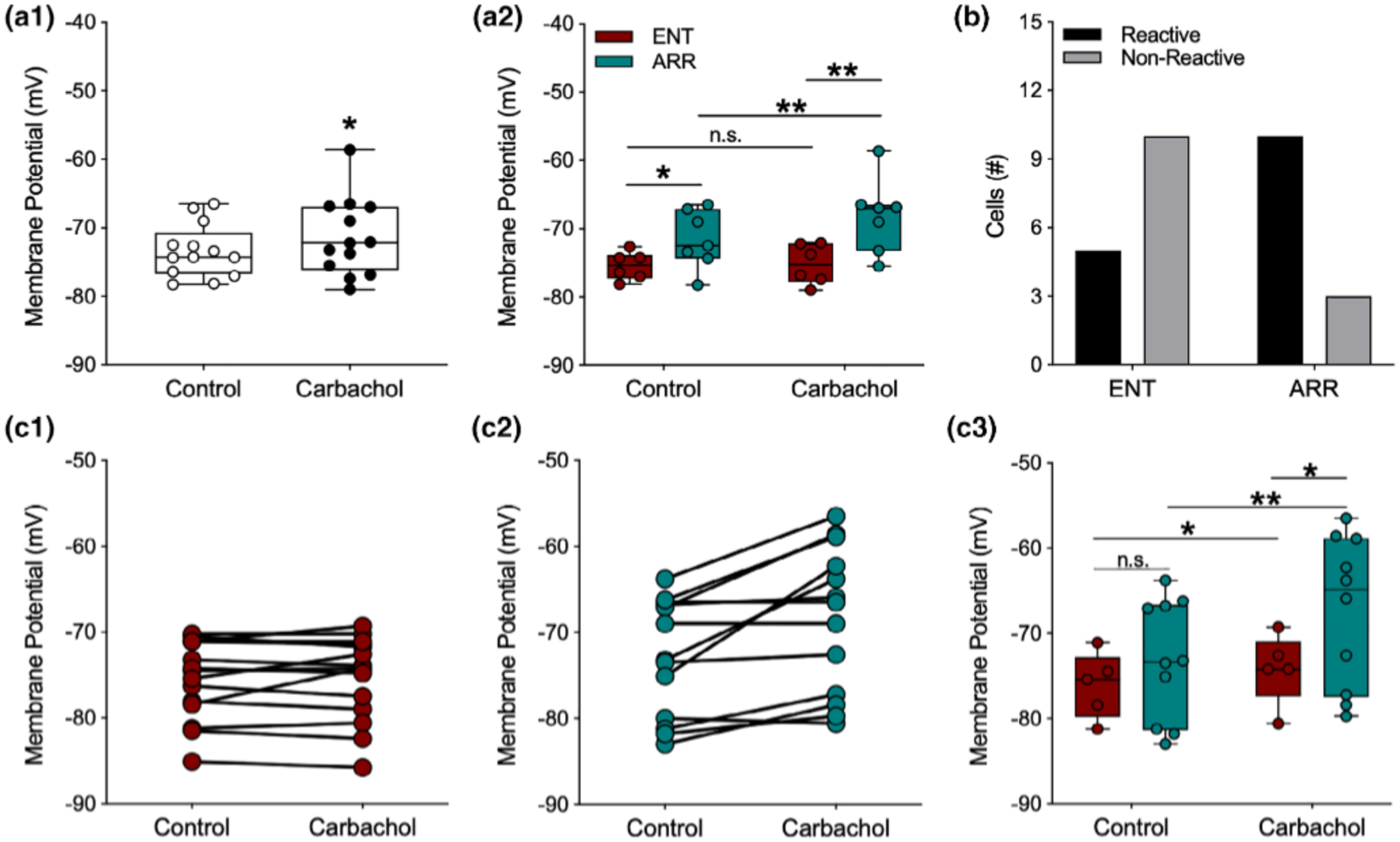
Membrane potentials of dentate granule cells were more depolarized by carbachol in arrhythmic animals. For data points in panel (a2) potentials were averaged across cells within individual animals so that each point is the mean value for a single animal; (a1, b), and (c1–c2) all represent individual cells. For panel (c3), only cells that depolarized under carbachol were analyzed. Carbachol (10 mM), caused significant but modest depolarization of the resting membrane potential (A1; *n* = 13). However, when data are separated by rhythm status, significant average depolarization only occurred in cells from ARR animals (a2; ENT, *n* = 6; ARR, *n* = 7). The resting potential of most ENT cells were not reactive to carbachol (b), while most cells in slices from ARR animals were reactive (b; Fisher’s exact test, *p* = .008). Membrane potentials of all individual neurons (c1, ENT, *n* = 15; c2, ARR, *n* = 13). Membrane potentials only of individual neurons that reacted (depolarized) in response to carbachol (c3). Box plot parameters as in Figure 4. *p < .05, **p < .01

Carbachol exerts several effects throughout hippocampal circuitry, both excitatory and inhibitory, particularly through its agonism at muscarinic receptors (Dannenberg et al., 2017). One well-known effect of activating muscarinic receptors in neurons of the hippocampal formation is that it causes slow depolarization of the resting membrane potential of neurons (Adams, Brown, & Constanti, 1982a, 1982b; Cole & Nicoll, 1984a, 1984b; Madison et al., 1987; Pitler & Alger, 1990). Thus, we were surprised to find that application of carbachol caused only a very small depolarization in dentate granule cells of 1.98 ± 0.88 mV, mean ± SE (ENT and ARR cells combined; resting: −73.4 mV ± 1.07; carbachol: −71.4 mV ± 1.6, *n* = 13, paired *t*-test, *p* = .043; Figure 5a1). However, when we compared ENT and ARR responses to carbachol, a surprising finding emerged: dentate granule cells from ARR animals depolarized, but ENT cells did not (carbachol: *F*_(1,11)_ = 5.73, *p* = .036; rhythm condition: *F*_(1,11)_ = 6.96, *p* = .023; Figure 5a2; ENT resting: −75.46 ± 0.83 mV, carbachol: −75.25 ± 1.25 mV; Δ 0.21 mV; *p* > .05, Sidak s; ARR resting: −72.86 ± 0.90 mV, carbachol: −68.46 ± 2.36 mV; Δ 4.40 mV; *p* = .014, Sidak’s; Figure 5a2). Furthermore, the resting membrane potential of ARR cells was significantly more depolarized than it was in cells from the ENT group (Δ 2.60 mV; *p* = .018, Sidak’s; Figure 5a2), and this difference was even greater under carbachol (Δ 6.79 mV; *p* = .008, Sidak’s; Figure 5a2). In fact, most of the ENT neurons did not depolarize (i.e., react) at all in the face of the carbachol challenge (Reactive; 5 of 15 cells), while most of the ARR neurons did depolarize (Reactive; 10 of 13 cells; Fisher’s exact test: *p* = .021; Figure 5b).

An examination of the effects of carbachol on the membrane potential of individual cells revealed that there was a strong average correlation between the resting membrane potential and the subsequent magnitude of carbachol-induced depolarization in individual neurons (Pearson, *r* = 0.87, *p* < .001, *n* = 28). However, examination of individual neurons within the ARR group (Figure 5c2) showed that neurons both in the more hyperpolarized and depolarized ranges of the distribution depolarized upon carbachol application, and that nonreactive cells in the ARR group were also found at both depolarized and hyperpolarized ends of the resting membrane potential distribution. This suggests that the value of the resting membrane potential alone cannot account for the differences in reactivity among cells from ENT and ARR groups. To test this possibility, only the carbachol-responsive neurons in each group (Figure 5c1,c2) were examined. We found that when nonresponsive cells were excluded, there was a strong main effect for carbachol on membrane potential (*F*_(1,13)_ = 17.16, *p* = .001) that was nearly three-fold greater among neurons from ARR animals (Δ mV, 5.78 ± 1.25, *n* = 10; *p* < .001, Sidak’s) than it was for ENT hamsters (Δ mV, 1.94 ± 0.72, *n* = 5; *p* = .045, Sidak’s; Figure 5c3). Furthermore, membrane potential under carbachol was more depolarized in ARR compared to ENT cells (*p* = .017, Sidak’s; Figure 5c3). Taken together, these findings suggest that the effect of carbachol on membrane potential arose from both the magnitude of depolarization and the proportion of reactive cells.

### 3.6 | Sex differences

Sex differences have been reported for several parameters of hippocampal function (Koss & Frick, 2017; Yagi & Galea, 2018), therefore, we performed post-hoc analyses of our data for differences among male and female hamsters. We found no sex differences in any of the experiments for I/O curves, PPF, or LTP performed in the dentate or in CA1. For clarity sake, statistical details of those negative findings are not presented.

**FIGURE 6:**
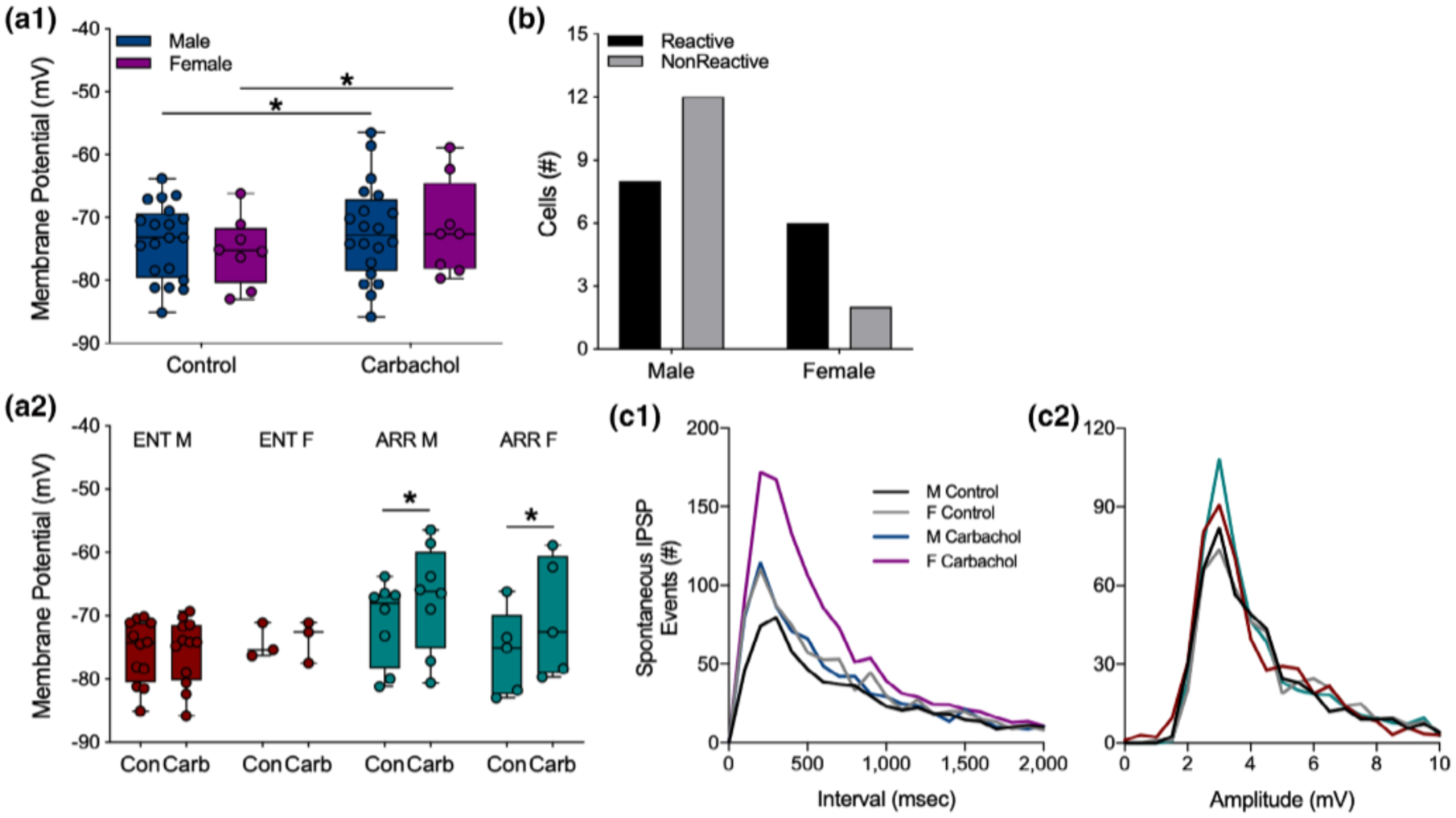
Sex differences in carbachol effects on dentate granule cells. Data from ARR and ENT animals were combined to show that overall, cells from both males and females depolarized under carbachol application (a1). When these data are parsed out by rhythm status (a2), no carbachol-induced depolarization was seen in either female or male ENT animals. Significant depolarization by carbachol was detected, however, in cells from both male and female ARR animals (a2). The resting membrane potential was greater in cells from male ARR compared to those from male ENT animals (a2). We found no significant sex differences in the proportion of cells that were reactive to carbachol (*p* > .05; b). Frequency distributions of spontaneous IPSP intervals (c1) and amplitudes (c2) during the first 150 s of recording. Statistical comparisons were made with KS tests from cumulative frequency distributions of the data in panels (c1) and (c2). There were no sex differences in control conditions (*p* > .05). Carbachol increased the number of spontaneous IPSP events in females (*p* = .021), but not in males (*p* > .05). There were no significant differences (*p* > .05) in amplitudes (c2) of spontaneous ISPS events. Box plot parameters as in Figure 4. *p < .05

Cells from both sexes depolarized in response to carbachol (*F*_(1,26)_ = 11.42, *p* = .002; Sidak’s: males, *p* = .049, *n* = 20; females, *p* = .047, *n* = 8; Figure 6a1). When we further separated the data by both sex and rhythm status, we found that cells from ENT males and ENT females did not depolarize in response to carbachol (*p* > .05 for both groups; Figure 6a2). By contrast, cells from ARR animals did depolarize in response to carbachol (*F*_(1,11)_ = 13.57, *p* = .003; Figure 6a2). Post-hoc tests revealed significant effects in both males (*p* = .047) and females (*p* = .033; Figure 6a2). There were no significant sex differences in the proportions of reactive and nonreactive cells (Fisher’s exact test, *p* > .05; Figure 6b).

Cumulative distributions were used to test for sex differences in the effects of carbachol on the frequency and amplitude of IPSP events. No sex differences were found in control recordings (KS test, *p* < .05). Application of carbachol increased the frequency of these events in females (KS test, *p* = .021), but not in males (KS test, *p* > .05; Figure 6c1). No sex differences were found in the amplitudes of these events (*p* > .05 for all KS tests of amplitude differences; Figure 6c2). There were no sex differences in evoked amplitude (all comparisons, *p* > .05; data not shown), nor were there sex differences in any other measures.

## 4 | DISCUSSION

Siberian hamsters are uniquely suited to studies of circadian rhythm effects on memory processing because their rhythms can be eliminated without modifications to their genome or surgical interventions. Elimination of circadian rhythms in these animals impairs spatial and recognition memory (Fernandez et al., 2014; Ruby et al., 2013). Because these forms of memory are hippocampus-dependent, we investigated hippocampal synaptic physiology *in vitro* in circadian-arrhythmic hamsters for a mechanism that could explain their memory deficits. In general, ARR and ENT groups did not differ in the basic physiological function of hippocampal synapses or synaptic circuitry, including LTP. However, we found that loss of circadian timing increased synaptic inhibition in dentate circuits in both control recordings and especially in response to the cholinergic agonist, carbachol. Thus, the loss of circadian timing that causes memory deficits also produced changes in hippocampal function that persisted in vitro in the absence of SCN influence.

Our focus on Ach was based on the well-established role for cholinergic projections from the medial septum/diagonal band to the dentate gyrus in spatial memory, and in particular for alternation behavior (Gold, 2003; Haam & Yakel, 2017; Ragozzino, Pal, Unick, Stefani, & Gold, 1998). Based on the role of Ach in spatial memory, we developed a working model to explain how the circadian clock in the SCN might influence cholinergic signaling in the dentate gyrus. Anatomical evidence from mice, rats, and hamsters indicates that the SCN projects to only one target in the entire limbic system-the ventrolateral septal nuclei (LSv; Watts, Swanson, & Sanchez-Watts, 1987; Morin, Goodless-Sanchez, Smale, & Moore, 1994; Kriegsfeld, Leak, Yackulic, LeSauter, & Silver, 2004). The main innervation target of the LSv is the medial septal nuclei (MS; Risold & Swanson, 1997), which is one of the major cholinergic regions in the brain (Dutar et al., 1995; Teles-Grilo Ruivo & Mellor, 2013). Cholinergic neurons here innervate the hippocampal formation in different regions, but the septohippocampal pathway into the dentate has been identified as a critical circuit for spatial memory and is essential for alternation behavior (Gold, 2003; Solari & Hangya, 2018).

By contrast to the anatomical evidence, functional evidence for an SCN-septal-dentate circuit is limited. Nevertheless, our behavioral data have shown that in its arrhythmic state, the SCN severely impairs alternation behavior, and that its subsequent ablation restores memory (Fernandez et al., 2014). The MS innervates the dentate with fibers that express Ach, glutamate, or GABA, but only the cholinergic projection is critical for alternation behavior. Signaling from the SCN to the LSv might be inhibitory given that nearly all SCN neurons synthesize and secrete gamma-aminobutyric acid (GABA; Moore & Speh, 1993; Albers, Walton, Gamble, McNeill 4th, & Hummer, 2017). Normally, firing rates of SCN neurons oscillate on a 24-hr rhythm with activity highest during the midday and lowest at night (Margraf, Puchalski, & Lynch, 1992; Mason, 1991; Ruby & Heller, 1996). In Syrian and Siberian hamsters, neurons in the arrhythmic SCN continue to discharge at the same average daily rate (Margraf et al., 1992; Mason, 1991). Thus, even in the arrhythmic state, the SCN is sufficiently active to influence its targets, but neuronal signaling would be continuous rather than rhythmic.

Several results from the present study support this model. First, we found that paired-pulse facilitation of population spikes was enhanced by the application of carbachol in both groups, but that this facilitation was markedly attenuated in cells from ARR animals. Second, the difference between ENT and ARR groups in carbachol-induced increase in facilitation was unlikely due to a difference in the amounts of presynaptic neurotransmitter release, because there was no carbachol-induced change in paired-pulse facilitation of postsynaptic EPSPs in either group. Therefore, we concluded that differences among ENT and ARR groups in the carbachol-enhancement of population spike facilitation must be modulated by some other postsynaptic mechanism.

One mechanism by which carbachol might exert its excitatory effects would be an indirect decrease in synaptic inhibition (Bell et al., 2013; McQuiston, 2014; McQuiston & Madison, 1999a, 1999b). Stimulus-evoked monosynaptic inhibition is significantly reduced by carbachol in cells from ENT animals, but not in those from ARR hamsters. We note that because these are monosynaptic-evoked IPSPs, the reducing action of carbachol is downstream from the excitatory parts of the local neuron circuit that normally activate the interneurons. In addition to the effects on stimulus-evoked inhibition, tonic spontaneous IPSPs occurred at a higher frequency in the ARR group. Although carbachol increased spontaneous IPSPs in both ENT and ARR tissues through muscarinic receptor activation, they reached a higher absolute level in cells from ARR animals. Thus, the loss of circadian timing was accompanied by an increase in the net inhibition in dentate circuits. This is consistent with the reduction in the paired-pulse facilitation of the population spike PPF observed in the ARR group compared to the ENT one. Ultimately, this would suggest that the output of dentate granule cells, and thus the passage of information to downstream circuits in the hippocampus, is reduced in ARR compared with ENT hamsters.

We further found that dentate granule cells from ARR animals not only rest at more depolarized potentials than those from ENT animals, but respond to carbachol in greater proportion and with a much greater increase in membrane potential. These effects may be due to the role of interneurons in modulating granule cell activity. The rate of spontaneous action potential outflow of interneurons is highly sensitive to their resting membrane potential, and these cells increase their discharge rates when they are in a more depolarized state (Bergles et al., 1996; Doze et al., 1991; McQuiston & Madison, 1999a, 1999b). If this difference in resting potential were to generalize to inhibitory interneurons, it could explain the difference in spontaneous IPSP rates that we observed. While we did not record directly from inhibitory interneurons in this study, it is well known that spontaneous IPSPs are highly sensitive to interneuron membrane potential, including the depolarizing action of muscarinic receptor activation (Bergles et al., 1996; Doze et al., 1991; McQuiston & Madison, 1999a, 1999b; Pitler & Alger, 1992). Thus, it is likely that inhibitory interneurons of ARR animals would be more strongly depolarized by carbachol.

We also found sex differences in two measures. The first is that males had a greater proportion of cells with membrane potentials that were non-reactive to carbachol, while cells in females were more likely to be reactive. The second finding was that the action of carbachol to increase spontaneous IPSP frequency was more pronounced in females than males. These data could be interpreted to imply that there was a greater net inhibition in female dentate circuitry compared to males. While there are documented sex differences in specific parameters of hippocampal function (Koss & Frick, 2017; Yagi & Galea, 2018), one must interpret these findings with caution and in the larger context of hippocampal circuitry. There may be other unknown sex differences that would offset the ones reported here resulting in no net sex differences in memory functions. We routinely examine our behavioral data for sex differences, but have found no such differences in performance on tests of spatial and recognition memory (Ruby et al., 2013).

## 5 | SUMMARY

Excitatory/Inhibitory balance of synaptic transmission is central to the function of any neural circuit. We found that circadian arrhythmia caused shifts in this balance in the intrinsic function of dentate granule cells and in their responses to muscarinic receptor activation. In general, excitatory transmission was modestly decreased by carbachol in both ENT and ARR animals, however, carbachol disinhibited the dentate circuitry in ENT animals while it increased net inhibition in ARR hamsters. Thus, the E/I balance of dentate gyrus circuitry was shifted toward excitation in ENT tissue, and conversely shifted toward inhibition in the dentate of ARR hamsters. This shift in E/I balance likely has important behavioral consequences, given that dentate granule cell mossy fibers project exclusively to hippocampal area CA3 (Prince, Bacon, Tigaret, & Mellor, 2016; Sasaki et al., 2018; Senzai, 2019), and that reduced excitation in the dentate-CA3 microcircuit impairs spatial memory (Flasbeck, Atucha, Nakamura, Yoshida, & Sauvage, 2018; Senzai, 2019). Thus, circadian-dependent differences in resting membrane potential and associated changes in rates of inhibitory events may explain the spatial and recognition memory deficits observed in circadian-arrhythmic hamsters.
